# Molecular Characterisation of *Mycobacterium bovis* Isolates from Cattle Slaughtered in Adamawa and Gombe States, North-Eastern Nigeria

**DOI:** 10.3390/cimb45070382

**Published:** 2023-07-19

**Authors:** Sadiq Mohammed Damina, David Atomanyi Barnes, Bitrus Inuwa, Gulak Hussaini Ularamu, Mohammed Bello, Olu Solomon Okaiyeto, Ayuba Caleb Kudi, Jeewan Thapa, Chie Nakajima, Yasuhiko Suzuki

**Affiliations:** 1 National Veterinary Research Institute, P.M.B.01, Vom 930103, Nigeria; 2 Division of Bioresources, Hokkaido University International Institute for Zoonosis Control, Kita 20 Nishi 10, Kita-ku, Hokkaido 001-0020, Japan; 3 Department of Veterinary, Medicine, Faculty of Veterinary Medicine, Ahmadu Bello University, Zaria 810211, Nigeria; 4 International Collaboration Unit, Hokkaido University International Institute for Zoonosis Control, Kita 20 Nishi 10, Kita-ku, Hokkaido 001-0020, Japan; 5 Division of Research Support, Hokkaido University Institute for Vaccine Research and Development, Kita 20 Nishi 10, Kita-ku, Hokkaido 001-0020, Japan

**Keywords:** bovine tuberculosis, Nigeria, *Mycobacterium bovis*, spoligotyping, MIRU-VNTR

## Abstract

Bovine tuberculosis is endemic in Nigeria with control measures as provided by the laws of the country being minimally enforced mostly at the abattoirs only. This study focused on bovine tuberculosis in Adamawa and Gombe States. Tuberculosis lesions were observed in 183 of 13,688 slaughtered cattle in the regions between June and December 2020. Analysis of tissue samples resulted in 17 *Mycobacterium bovis* isolates, predominantly from Gombe State. Spoligotyping identified four spoligotypes, including SB0944, SB1025, SB1104, and one novel pattern. MIRU-VNTR analysis further differentiated these spoligotypes into eight profiles. All isolates belonged to the Af1 clonal complex. The study emphasises the need for broader coverage and more isolates to comprehensively understand the molecular epidemiology of bovine tuberculosis in Nigeria. To enhance research and surveillance, a cost-effective approach is proposed, utilising a discriminatory VNTR panel comprising five or nine loci. The five-locus panel consists of ETR-C, QUB26, QUB11b, MIRU04, and QUB323. Alternatively, the nine-locus panel includes ETR-A, ETR-B, QUB11a, and MIRU26. Implementing this approach would provide valuable insights into the genetic diversity of *M. bovis* strains in Nigeria. These findings are crucial for developing effective control measures and minimising the impact of bovine tuberculosis on both animal and human health.

## 1. Introduction

The genus *Mycobacterium* includes over 200 different species that includes approximately 30 causing a number of human and animal diseases such as tuberculosis, non-tuberculous mycobacterial diseases and leprosy [1]. Bovine tuberculosis (bTB) which is caused primarily by *Mycobacterium bovis* but also by *Mycobacterium caprae*, is a highly significant zoonotic disease that can be transmitted either through aerosols or consumption of unpasteurised milk and other dairy products [2]. *M. bovis* is a member of the *Mycobacterium tuberculosis* complex (MTBC) which also includes *M. tuberculosis, Mycobacterium africanum, Mycobacterium caprae, Mycobacterium pinnipedii, Mycobacterium canetti, Mycobacterium microti, Mycobacterium mungi, Mycobacterium orygis, Mycobacterium suricattae* and the Dassie bacillus [3]. bTB has been known to cause serious economic losses that arise from the cost of eradication programmes, trade restrictions and the threat the disease poses to both endangered and protected species as well as its public health implications [4]. Across the world, the control and eradication of bovine tuberculosis can be complicated by wildlife reservoirs of the disease especially in countries with large populations of wildlife [5,6].

The occurrence of bTB has been reported in virtually all parts of Africa despite the paucity of knowledge about the distribution, prevalence, and epidemiological patterns of the disease in the continent [7]. The disease is an endemic zoonosis of serious public health concern in Africa and in most parts of the continent control of bovine tuberculosis is hampered by several unfavourable socioeconomic conditions such as co-infection with HIV [8]. The disease is of particular importance to many of the countries in Africa especially those in arid and semi-arid regions where it is reported that more than 50% of all African cattle, sheep and goats are kept and where most of the people depend on livestock for their livelihood [7]. The heaviest burden of bovine tuberculosis is found on poor marginalised rural communities that live in close contact with their livestock and have poor access to safe food and health care [9].

Limited information is available on the epidemiology of bovine tuberculosis in Nigeria especially the molecular epidemiology aspect [10]. The level of transmission of tuberculosis between animals and humans in Nigeria is not well known but there are cultural practices that are believed to favour the transmission of the disease from cattle to humans and these include the raising and fattening of cattle around farmers’ homes and the slaughtering of and processing of these animals in nearby abattoirs with minimal protection [11].

The overall prevalence of bovine tuberculosis in Nigeria is currently not known but studies in different parts of the country have revealed varying reports. In Bauchi State, for instance, a prevalence of 15%, 29.16% and 8.33% by post-mortem, Ziehl–Neelsen and Polymerase Chain Reaction (PCR), respectively, was reported [12]. A study conducted in 2018 [13] at selected abattoirs in Bauchi and Gombe States reported the prevalence of bovine tuberculosis to be 9.8% and 13.9%, respectively, while a 5-year retrospective study in Adamawa State, Northeastern Nigeria reported a prevalence of 6.7% [14] and this was higher than the prevalence of 0.34% reported in 2009 [15] for Adamawa State and 2.8% for Damaturu (Yobe State), Maiduguri and Damboa (Borno State) in 2001 [16]. An overall prevalence of 8.2% of bovine tuberculosis was reported in Rivers State, Southern Nigeria in cattle slaughtered in the University of Portharcourt central abattoirs where *M. bovis* was confirmed to be the causative agent of lesions observed [17]. The study also revealed that the lung was the most frequently affected organ by the disease where 43.2% of tuberculous lesions were detected in the lungs during postmortem examination [17]. In northwestern Nigeria, a prevalence of 5% was reported in Kaduna State following tuberculin skin testing and 1.7% by Ziehl–Neelsen testing of milk samples of purified protein derivatives (PPD) positive cows [18]. In Plateau State, north central Nigeria the prevalence of bovine tuberculosis was reported to be 4.4% based on detection of tuberculous lesions in slaughtered cattle at abattoirs [19]. In Enugu State, southeastern Nigeria the prevalence of tuberculosis in cattle based on abattoir records was reported to be 1.4% [20].

Understanding the transmission dynamics and sources of infection of tuberculosis heavily relies on epidemiological studies, which are also important tools for comprehending the risk factors associated with the disease and tracing back its sources of infection. In Cameroon, a country that shares international borders with Adamawa State, northeastern Nigeria, bovine tuberculosis has been reported to be prevalent in slaughtered cattle at abattoirs with 81.53% and 48.82% of carcass condemnations in two major districts being due to tuberculosis [8]. These findings underscore the significance of epidemiological studies in understanding the transmission of tuberculosis and tracing back sources of infection. Moreover, genotyping tools are indispensable in unravelling the intricate transmission patterns and dynamics of the disease, as well as shedding light on the risk factors involved [21].

Bovine tuberculosis is a major problem to most developing countries, especially those in sub-Saharan Africa including Nigeria, yet it remains a neglected zoonosis of serious public health importance. Information on the epidemiology of the disease especially with regards to circulating strains of *M. bovis* in cattle population is scanty in Nigeria.

This study is therefore designed to determine the prevalence of bovine tuberculosis in Adamawa and Gombe States, northeastern Nigeria, and to identify *M. bovis* strains circulating in cattle presented for slaughter at selected abattoirs in the two states.

## 2. Materials and Methods

### 2.1. Study Areas

**Figure 1:**
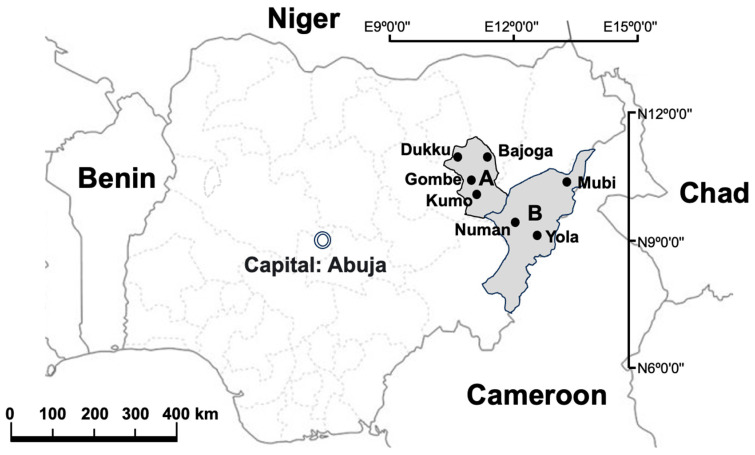
Map of Nigeria showing the neighbouring countries as well as the sample study sites. A: Gombe State; B: Adamawa State. ArcGIS 10.2.2 Software esri, available online: https://www.esri.com (accessed on 12 July 2023).

The study was conducted in Adamawa and Gombe States, northeastern Nigeria, where samples were collected at selected abattoirs and slaughterhouses in the two states between June 2020 and December 2020. The sampling sites in Gombe State were Gombe abattoir (N10°17′32″ E11°11′26″), Bajoga slaughterhouse (N10°52′20″ E11°25′49″) Dukku slaughterhouse (N10°49′26″ E10°45′21″) and Kumo slaughterhouse (N10°1′53″ E11°12′22″). In Adamawa state, the sampling sites were Yola abattoir (N9°12′51″ E12°27′4″), Mubi slaughterhouse (N10°16′8″ E13°17′7″) and Numan slaughterhouse (N9°27′43″ E12°1′12″) (Figure 1).

The selection of these abattoirs and slaughterhouses was based on the fact that they are the main slaughter sites for cattle in the two states. Most of the cattle slaughtered in these abattoirs and slaughterhouses come from different cattle markets and individual farms located across the two states as well as from other northeastern states of Nigeria and some neighbouring countries such as Niger, Chad and Cameroon (Figure 1)。

### 2.2. Sample Collection and Culture

The permission and approval of the directorates of veterinary services of the respective states was sought before the commencement of sampling for the study. The target population for this study was cattle observed with gross lesions compatible with tuberculosis during meat inspections at the different abattoirs and slaughter slabs.

Detailed post-mortem examination of carcasses involving examination, palpation, and incision of the various organs for lesions suggestive of tuberculosis was performed on all slaughtered cattle during meat inspections. Tissues with lesions compatible with tuberculosis were collected using sterile scissors and forceps into clean, sterile, and properly labelled plastic specimen containers with top screw caps. Samples were collected, labelled, and transported on ice packs in durable, leak proof containers to the laboratory for analysis. The samples were collected from animals slaughtered and examined at the different abattoirs and slaughter slabs for a period of seven months. Due to the distance between sample collection sites and the laboratory, samples were frozen for between one and two months before processing.

Decontamination, culture, and isolation were carried out according to the modified Petroff method where 4% NaOH was used as a digestant and decontaminant. Briefly, the samples were homogenised using a tissue grinder (Kisker Biotech & Co KG, Steinfurt, Germany) and 4 mL of 4% NaOH added and the mixture vortexed (Vortex Genie^®^2, Scientific Industries, Bohemia, NY, USA) and allowed to stand for 15 min at room temperature. The tubes were filled with PBS and centrifuged at 3000 rpm (MX 205, TOMY, Tokyo, Japan), 4 °C for 20 min using a refrigerated centrifuge. The supernatant was decanted and the sediments resuspended in 1 mL of PBS and 0.5 mL was used for inoculating 2 Löwenstein–Jensen (LJ) tubes, one containing glycerol and the other one containing pyruvate. The LJ tubes were incubated at 37 °C for 8 weeks with observation for growth on a weekly basis.

### 2.3. DNA Extraction and Mycobacterial Species Confirmation

DNA extraction from all samples was carried out at the National Veterinary Research Institute, Vom using a QIAamp^®^ DNA Mini Kit following the manufacturer’s instructions. The multiplex PCR assay described by [22,23] with minor modifications was used to confirm the isolates as *M. bovis*. The reaction was carried out in a 20 µL total volume containing 4 µL of 5× Go Taq buffer (Promega Co., Madison, WI, USA), 0.8 µL of 25 mM MgCl_2_, 2 µL of 2.5 mM dNTP mix, 2 µL of 5 M Betaine, 0.5 µL of 10 µM each primer targeting the region of difference (RD4) and double-distilled water (DDW). The common forward primer used was CBS1 (5′-TTCCGAATCCCTTGTGA-3′), while the *M. bovis*-specific reverse primer was CBS2 (5′-GGAGAGCGCCGTTGTA-3′) and the *M. tuberculosis*-specific reverse primer was CBS3 (5′-AGTCGCCGTGGCTTCTCTTTTA-3′). GoTaq DNA polymerase (5 U/µL, Promega Co.) and 1 µL of template DNA were also added. The cycling conditions were as follows: initial denaturation at 96 °C for 1 min, 35 cycles of denaturation at 96 °C for 10 s, annealing at 50 °C for 30 s and extension at 72 °C for 30 s, with a final elongation step at 72 °C for 3 min. The expected PCR products were 337 bp for *M. tuberculosis* and 168 bp for *M. bovis*.

### 2.4. Molecular Typing

Spoligotyping and Mycobacterial Interspersed Repetitive Unit-Variable Number Tandem Repeat (MIRU-VNTR) were performed at the Hokkaido University International Institute for Zoonosis Control, Japan.

Spoligotyping was as described by [24,25]. Initially, the direct repeat (DR) region of *M. bovis* was amplified via PCR using a primer pair designed specifically for the direct repeat sequence. The amplified PCR products were then subjected to hybridisation with a specialised membrane that contained 43 unique oligonucleotide probes, each corresponding to a distinct spacer within the DR region. The presence or absence of spacers in the PCR products was represented by ‘1’ or ‘0’, respectively. Consequently, an octal code number was derived from the spoligotyping pattern. This code number was subsequently entered into the online database https://www.mbovis.org/database.php accessed on 23^rd^ November 2022, which facilitated the generation of the corresponding SB number for further analysis and comparison.

MIRU-VNTR was carried out according to the method described by [26] with a slight modification (QUB4156, Mtub29 and Mtub34 were replaced with QUB3232, ETR-F and QUB11a) to improve discriminatory power using primers for 24 MIRU-VNTR loci. The reaction was performed in a thermal cycler beginning with an initial denaturation at 95 °C for 3 min followed by 35 cycles of 95 °C for 15 s, 55 °C for 20 s and 72 °C for 45 s and a final extension at 72 °C for 5 min. The primer annealing temperature was changed to 50 °C for loci QUB11b and MIRU4. Electrophoresis was performed on a 2% agarose gel prepared in 0.5% TBE buffer and a 50 bp DNA ladder was used in determining the size of amplicons of the PCR products and visualisation was carried out under UV light.

The discriminatory power of the 24 Loci MIRU-VNTR was assessed using the Hunter-Gaston Discriminatory Index (HGDI) [27,28]. It takes into account the total number of studied isolates (*N*), the number of isolates with a specific genetic pattern (*nj*), and the total number of distinct genetic patterns observed (*s*). The HGDI is calculated using the formula:

$$HGDI=1-\frac{1}{N\left(N-1\right)}\sum_{j=1}^{S}nj\left(nj-1\right)$$

Loci with high allelic diversity (HGDI > 0.6) are considered highly discriminatory, indicating a substantial level of genetic variability and the ability to distinguish between different isolates. Loci with moderate allelic diversity (0.3 < HGDI < 0.6) exhibit a moderate level of discriminatory power, while loci with low allelic diversity (HGDI < 0.3) are considered poorly discriminatory.

The MIRU-VNTR profiles obtained from the isolates were subjected to analysis, and a dendrogram was subsequently constructed using the MIRU-VNTR*plus* platform, employing the unweighted pair group method with arithmetic mean (UPGMA) algorithm. The UPGMA method utilised data from the 24 loci and spoligotyping patterns to represent the relationships between isolates based on distance matrices.

## 3. Results

Out of a total of 10,974 and 2714 animals slaughtered in Adamawa and Gombe States, respectively, 101 in Adamawa and 82 in Gombe had lesions compatible with tuberculosis. Of all the 183 tissue samples cultured on LJ media supplemented with glycerol and pyruvate, 17 isolates were recovered with 11 on glycerol and 6 on pyruvate-supplemented media. Out of the 17 isolates, only 2 were from Adamawa, the remaining 15 were from samples collected in Gombe State (Table 1).

All 17 samples analysed in this study were confirmed to be *M. bovis* using the multiplex PCR assay. The presence of the expected band size of 168 bp, specific to *M. bovis*, was observed in each sample, validating their identification.

**Figure 2:**
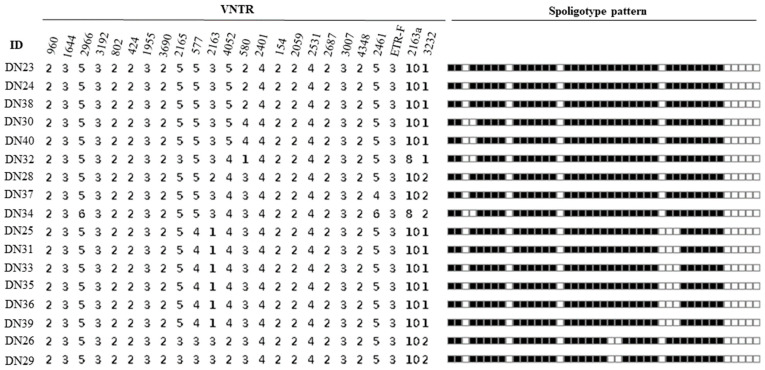
Spoligotype and MIRU-VNTR profiles of isolates.

The spoligotyping pattern and the number of repeats for each of the MIRU-VNTR for all the 17 isolates is as shown in Figure 2. Spoligotyping analysis served as additional evidence to confirm that all isolates belonged to *M. bovis*, as they lacked the characteristic spacers 3, 9, 16, and 39–43 of *M. bovis* strains (Figure 2). It differentiated the 17 isolates into 3 known spoligotypes: SB0944, SB1025, SB1104, and 1 unidentified pattern. Among these, SB0944 and SB1025 were identified as the predominant strains. In total, 13 isolates from Gombe shared known spoligotype patterns, while the remaining 2 displayed the unique, unidentified pattern. In contrast, the 2 isolates from Adamawa displayed spoligotype patterns consistent with SB0944 and SB1025.

**Figure 3:**
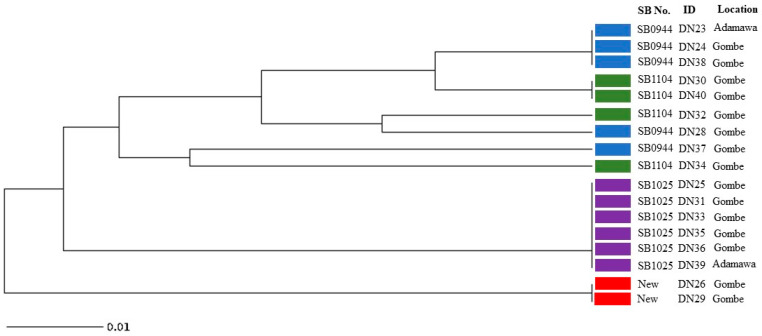
UPGMA tree phylogenetic analysis of *Mycobacterium bovis* isolates from cattle slaughtered in Adamawa and Gombe states revealing relationships based on 24 MIRU-VNTR loci and spoligotype patterns (Blue: SB0944; Green: SB1104; Purple: SB1025; Red: New spoligotype pattern).

The 4 spoligotype patterns identified in this study were further characterised into 8 distinct VNTR profiles (Figure 3). The results obtained from analysing the MIRU-VNTR loci are presented in Table 2, which includes information on the locus alias, observed tandem repeat numbers for each allele, allelic variants (AV), and allelic diversity (HGDI) for each locus. The allelic diversity, represented by HGDI values ranging from 0 to 0.56, reflects the level of genetic diversity at each locus. Among the analysed MIRU-VNTR loci, ETR-C exhibited the highest allelic diversity with a value of 0.56. QUB26, QUB11b, MIRU04, and QUB3232 followed in descending order, displaying moderately discriminatory indices ranging between 0.3 and 0.6. On the other hand, ETR-A, ETR-B, QUB11a, and MIRU26 demonstrated a poor discriminatory index (HGDI < 0.3). The remaining loci exhibited limited genetic variation and were unable to effectively discriminate the isolates in this study.

## 4. Discussion

Bovine tuberculosis is endemic in Nigeria and constitutes a serious public health challenge in the country with the application of few or no preventive measures [10]. Virtually all the animals examined in this study were owned and managed by the Fulani nomadic pastoralists who manage their cattle exclusively on an extensive management system. The nomadic pastoralists also own the greater proportion of cattle not only in Adamawa and Gombe States but also in Nigeria where they are known for their seasonal migration in search of greener pastures for their livestock [29]. Their migration is usually from the drier north with a scarcity of pasture and water to the south where there is abundant pasture and water. Consequent to this, animals from different herds can meet in the same grazing and watering areas. The nomads graze their livestock freely in the fields, crossing states and international boundaries thereby aiding in the transmission and spread of infectious diseases such as bovine tuberculosis [29]. Unfortunately, most of the herders are not aware of bovine tuberculosis and its zoonotic implications and in most instances, raw milk is one of their staple foods in the households.

There are three major cattle markets in Adamawa State from where butchers source their animals for slaughter and these include Mubi international cattle market, Ganye international cattle market and Ngurore cattle market. Mubi and Ganye international cattle markets are located in local government areas that share borders with the Republic of Cameroon (Figure 1) and animals and their products move across the borders freely [14]. However, there are other smaller markets from where animals can also be bought and taken to the abattoir for slaughter. Cattle markets in Gombe State include the Gombe, Kuri, Dadin kowa, Kashere, Kumo, Dukku, Bajoga, Laggal, and Kurugu cattle markets spread across the state. None of the cattle markets in Gombe State is located at the international border as the state only shares borders with other states in Nigeria including Adamawa State but it is pertinent to state that livestock and their products move freely across the country without any restriction. This means diseases including bovine tuberculosis can easily be spread from one point to another through traded livestock, livestock products, people, and even transportation vehicles.

During meat inspections at the abattoirs and slaughterhouses, organs with lesions suggestive of tuberculosis are removed and condemned while the rest of the carcass is passed for human consumption and this could be a potential source of zoonotic tuberculosis [14]. Abattoir workers and butchers also handle carcasses with little or no protective measures during the dressing of carcasses and meat inspections.

Following culture on Lowenstein–Jensen media supplemented with both glycerol and pyruvate, only 17 isolates were recovered, 2 from Adamawa and 15 isolates from samples collected in Gombe State. The low recovery of mycobacteria from the tissues could be attributed to the break in freezing temperatures during transportation from Adamawa and Gombe to Vom in Plateau State for processing after samples had been frozen for about 3 months in between. Out of the 17 isolates, 11 were obtained on glycerol-supplemented media while the other 6 were recovered from pyruvate-supplemented media.

All the 17 identified isolates were confirmed to be *M. bovis* by a multiplex PCR assay and spoligotype signatures, with the absence of spacer 3, 9, 16, and 39 to 43 characteristic of *M. bovis* spoligotypes (Figure 2). This suggests *M. bovis* to be the main cause of bovine tuberculosis in this study. With spoligotyping, the 17 isolates were differentiated into 4 spoligotype patterns; SB0944, SB1025, SB1104 and 2 isolates that shared an identical new pattern. All the 17 isolates were lacking in spacer 30 which is a common feature of the Africa1 (Af1) clonal complex of *M. bovis* that was reported to occur in high frequency in Mali, Chad, the Republic of Cameroon and Nigeria [30]. It is interesting to note that the newly identified spoligotype pattern indicates a possible evolutionary relationship with SB0944, as it lacks spacers 23 and 24. This finding suggests that the new pattern may have emerged through local evolution within Nigeria. Spoligotype patterns SB0944 and SB1025 were the dominant strains identified (Figure 3) and these were also reported in previous studies in southwestern Nigeria, the Republic of Cameroon, Chad and Mali in 2009 [30] while [31] also reported SB0944, SB1025 and SB1104 in the Republic of Cameroon which is a country that shares borders with Adamawa State, suggesting an existing cycle of transmission of these strains in these west central African countries. Spoligotypes SB0944 and SB1025 have also been observed in Borno State, northeastern Nigeria [32]. That the same strains observed in this study were observed in southwestern Nigeria is not surprising as most of the cattle slaughtered in the southern part of Nigeria are reared and brought from the northern part of the country where the greater proportion of the cattle in Nigeria are found. However, there are only few states out of the 36 states and Federal Capital Territory in Nigeria where molecular characterisation of *M. bovis* strains circulating in cattle has been carried out; therefore, the true picture of the different *M. bovis* strains in the entire country may not be clear now.

For Adamawa and Gombe State, this may also be the first study that involves characterisation of *M. bovis* strains using spoligotyping and MIRU-VNTR. The MIRU-VNTR profile analysis provided additional classification of the four spoligotype patterns into eight distinct subtypes. Interestingly, most of the loci examined exhibited no variation among the isolates, indicating a limited genetic diversity within these regions for these isolates. Notably, the spoligotype SB0944 demonstrated further differentiation, giving rise to three subtypes based on their VNTR patterns (Figure 3). All the VNTR subtypes of SB0944 displayed a closer genetic relationship to SB1104, suggesting potential evolutionary connections and genetic similarities between SB0944 and SB1104. It is also noteworthy that all isolates belonging to the SB1025 spoligotype, which was the most abundant spoligotype pattern among the isolates, and exhibited an identical VNTR pattern. This observation indicates a clonal expansion of the SB1025 strain, suggesting a common source or transmission event for these isolates. The two isolates displaying the unique and unidentified spoligotype pattern were found to share the same VNTR results pattern. This concordance in VNTR profiles suggests a close genetic relationship between these isolates, indicating a possible clonal origin or transmission event. Among the 24 MIRU-VNTR loci analysed, ETR-C exhibited the highest allelic diversity with a value of 0.56, indicating a wide range of genetic variation within this locus. QUB26, QUB11b, MIRU04, and QUB3232 followed closely behind, showing progressively decreasing allelic diversity values. These loci contributed significantly to the overall genetic diversity observed in the studied isolates. Considering that the tested samples were limited to 17, it is understandable that the allelic diversity, as represented by the HGDI values, may not reach high levels. However, it is important to note that the moderate discriminatory indices obtained from these loci still provide useful information for differentiation, thus we can recommend using these 5 discriminatory loci (ETR-C, QUB26, QUB11b, MIRU04, and QUB3232) as well as ETR-A, ETR-B, QUB11a, and MIRU26 (which had a low HGDI index) (Table 2) for PCR-based differentiation of Nigerian M. bovis strains. By utilizing these few loci based on our study we can distinguish between isolates based on their genetic profiles, even with a limited sample size.

The knowledge and proper understanding of the different strains of *M. bovis* circulating in these states and Nigeria as a whole is essential in understanding the transmission patterns of bovine tuberculosis and also in tracing back sources of infection and possible ways of controlling the disease in the country. The test and slaughter policy which has been adopted and used in the control of bovine tuberculosis in developed countries is not applied in Nigeria, similar to most other African countries. Control measures against zoonotic tuberculosis are only undertaken at the abattoir level during meat inspections where organs with tuberculous lesions are removed and condemned while the rest of the carcass is passed to the public for consumption.

Combining MIRU-VNTR with spoligotyping and other conventional methods for diagnosing and studying the transmission patterns of bovine tuberculosis is crucial for comprehending the disease situation among cattle populations in Nigeria. However, the lack of availability and affordability of these essential diagnostic tools hinders their widespread implementation in Nigeria and other developing countries. To overcome these challenges, a cost-effective approach utilising a subset of MIRU-VNTR loci is recommended. In particular, the utilisation of a discriminatory VNTR panel consisting of ETR-C, QUB26, QUB11b, MIRU04, and QUB323, or alternatively including ETR-A, ETR-B, QUB11a, and MIRU26, can provide valuable insights into the molecular epidemiology of bovine tuberculosis in Nigeria. These selected loci have demonstrated the ability to differentiate between genetic patterns while being more feasible in terms of resources and cost. Additionally, it is crucial to expand the scope of future studies beyond the current coverage of only 2 states out of the 36 states and FCT in Nigeria. By including additional regions, a more representative and inclusive assessment of the molecular epidemiology of bovine tuberculosis can be achieved. This expanded research approach will enhance our understanding of the disease dynamics across the country and facilitate the development of effective strategies for disease management and control in the cattle population of Nigeria.

## 5. Conclusions

In conclusion, this study reveals significant findings regarding the *M. bovis* strains in Nigeria, with all isolates identified as belonging to the Af1 clonal complex. However, due to the limited coverage of two states and a small number of isolates, it is crucial to expand the research to include all states and the Federal Capital Territory in order to obtain a comprehensive understanding of the molecular epidemiology of bovine tuberculosis in the country. To enhance research and surveillance efforts, we propose the utilisation of a discriminatory VNTR panel consisting of either five or nine loci. The five-locus panel includes ETR-C, QUB26, QUB11b, MIRU04, and QUB323, while the nine-locus panel additionally includes ETR-A, ETR-B, QUB11a, and MIRU26. By implementing this approach, valuable insights into the genetic diversity of *M. bovis* strains in Nigeria can be obtained in a cost-effective manner. Furthermore, this will contribute to the development of effective control measures for bovine tuberculosis, ultimately reducing its impact on both animal and human health.

## Figures and Tables

**Table 1:** Isolation of Mycobacterium bovis from cattle at Adamawa and Gombe slaughterhouses.

State	No. of Animals Slaughtered	No. of Animals with Lesions	% with Lesions	No. of Isolates Obtained	% of Isolates Obtained	No. of Isolates Confirmed as *M. bovis*
Adamawa	10,974	101	0.92	2	1.98	2
Gombe	2714	82	3.02	15	18.29	15
Total	13,688	183		17		17

**Table 2:** Allelic diversity values for each of the 24 MIRU-VNTR loci analysed in the 17 *M. bovis* isolates.

Locus	Alias	Number of Tandem Repeats											AV	Allele Diversity
		0	1	2	3	4	5	6	7	8	9	10		
577	ETR-C				2	6	9						3	0.56
4052	QUB26			2		10	5						3	0.53
2163	QUB11b		6	1	10								3	0.5
580	MIRU04		1	3	11	2							4	0.5
^a^	QUB3232		12	5									2	0.38
2165	ETR-A				3		14						2	0.25
2461	ETR-B					1	15	1					3	0.17
^a^	QUB11a									2		15	2	0.16
2966	MIRU26						16	1					2	0.06
960	MIRU10			17									1	0
1644	MIRU16				17								1	0
3192	MIRU31				17								1	0
802	MIRU40			17									1	0
424	VN424			17									1	0
1955	VN1955				17								1	0
3690	VN3690			17									1	0
2401	VN2401					17							1	0
154	MRU02			17									1	0
2059	MIRU20			17									1	0
2531	MIRU23					17							1	0
2687	MIRU24			17									1	0
3007	MIRU27				17								1	0
4348	MIRU39			17									1	0
^a^	ETR-F				17								1	0

^a^ non-standard 24 loci by [24].

## Data Availability

The data presented in this study are available on request from the corresponding author.
